# Physical functionality two years after major trauma: predictors from a single center cohort study

**DOI:** 10.1007/s00068-026-03255-9

**Published:** 2026-07-06

**Authors:** Katharina Fetz, Jan Koetsenruijter, Daniela Rodrigues Recchia, Johanna Rutetzki, Rolf Lefering, Sigune Kaske

**Affiliations:** 1 Institute for Research in Operative Medicine, Witten/Herdecke University, Ostmerheimer Str. 200, 51109 Cologne, Germany; 2 Chair of Research Methodology and Statistics, Witten/Herdecke University, Alfred-Herrhausen-Straße 50, 58455 Witten, Germany; 3 Medical Faculty, Heinrich Heine University, Duesseldorf University Hospital, Moorenstraße 5, 40225 Duesseldorf, Germany; 4 Department of Health and Safety, AXA Concern AG, Colonia-Allee 10-20, 51067 Cologne, Germany; 5 Chair of Quality of Life, Spirituality and Coping, Witten/Herdecke University, Gerhard-Kienle-Weg 4, 58313 Herdecke, Germany

**Keywords:** Major trauma, Physical functionality, Functional recovery, Pain, Health-related quality of life, Patient reported outcomes (PROM (s))

## Abstract

**Purpose:**

Traumatic injuries are a major global public health concern, often leading to impaired physical function and disability. Long-term physical function impairments can greatly affect a patient’s health related quality of life, influencing their emotional state and social interactions. This study examines patient-reported physical functionality before and after major trauma, identifying key predictors of poor functional recovery.

**Methods:**

This retrospective single-center cohort study investigates physical functionality 23 months after a traumatic injury using standardized questionnaires combined with clinical data. Inclusion criteria were Injury Severity Score (ISS) ≥ 9, requiring ICU treatment, and age ≥ 18 resulting in an eligible sample of 515 patients. Health outcomes were assessed using the Trauma Outcome Profile (TOP), the Abbreviated Injury Scale (AIS), and the ISS.

**Results:**

Physical functionality significantly decreased from 94.8 (SD 11.5) to 72.9 (SD 26.7) 23 months post-trauma with almost half of participants still scoring below the critical cutoff of 80 points. The strongest predictor for functional physical impairment was pre-trauma physical functionality (OR 8.21, *p* < 0.001). Other determinants included age (age 50–59 versus 18–29 OR 2.75, *p* = 0.001), as well as an impacted lower extremity including pelvic (OR 1.60, *p* = 0.018).

**Conclusion:**

The most important predictors for a post-trauma impaired physical function are pre-trauma functionality and age. Among trauma related factors, injuries involving the lower extremities remained significantly associated with poorer long-term outcomes, whereas overall injury severity was not independently associated with functional impairment.

## Introduction

Traumatic injuries remain a leading cause of death and long-term disability worldwide, particularly among younger adults [1–3]. Road accidents, falls, and violence are common causes and account for more than 10% of global deaths among individuals aged 5–44 [2]. Among survivors the most frequent problems are endured chronic pain, reduced mobility, psychological distress, and substantial decreases in life quality, affecting in this way not only the affected individuals but entire families [1, 4]. The economic burden from medical costs, ongoing rehabilitation, and loss of employment is significant [3]. Therefore, effective strategies in prevention and recovery management for survivors remain crucial [5].

Survival rates after trauma have improved, but many patients report for years after the initial injury bodily and emotionally restrictions and complaints, including physical disabilities, chronic pain, depression, anxiety, and post-traumatic stress disorder (PTSD) [6–9]. A full functional recovery in severely injured patients is unfortunately often limited and functioning remains below typical population levels [10–12]. Persistent pain is an aggravating factor, affecting 50–63% of survivors, and significantly limits physical activity while at the same time negatively impacting the overall quality of life [7, 8, 13–15]. Pain frequently coexists with psychological conditions, further complicating recovery [7, 11, 16, 17].

For these patients, returning to work is often difficult. Many survivors require prolonged recovery periods, face reduced employment opportunities, or lose their jobs entirely [8, 11]. Critical factors influencing employment include injury severity, injuries of the lower limbs or spine, pain intensity, mental health status, and socioeconomic characteristics [15, 18]. Compensable injuries in particular lead to extended recovery durations and increased bureaucratic challenges, negatively impacting functional outcomes [11].

For this reason, it is essential that a comprehensive rehabilitation program is developed in order to improve functional recovery and employment outcomes [7, 13, 18, 19]. In order to develop such a program, the factors that contribute to a functional recovery should be well understood. This study aims to examine patient-reported physical functionality before and after major trauma, identifying key predictors of poor functional recovery. We hypothesized that long-term patient-reported physical functionality after major trauma depends on patients’ baseline characteristics and on the severity of the injury caused by the trauma.

## Methods

### Study design

A retrospective single-center cohort study was developed in survivors of a traumatic injury. Standardized questionnaires were employed as a self-administered paper-and-pencil process approximately 23 months post-trauma combined with clinical data from individual patient injuries and their severity. The aim is to identify possible predictors associated with an impaired physical function two years after trauma. The data collection was approved by the Ethics Committee of Witten/Herdecke University (reference number: 20/2010). It adhered to the principles outlined in the Declaration of Helsinki, and participants were explicitly informed of their right to withdraw from the study at any time without any consequences.

### Setting and study population

The data used is based on an initial cohort of 681 patients treated at the Cologne Merheim Medical Center, an accredited level one trauma center, between 2008 and 2018. Out of a total number of about 2200 treated patients in this time period, this cohort contains those patients who returned a questionnaire on quality-of-life about 2 years post-trauma. Exclusion criteria for being included in this cohort were dead (either due to trauma or within two years post-trauma), a vegetative state (as indicated by a Glasgow Outcome Scale (GOS) score of 2), severe cognitive impairments preventing completion of the questionnaire (attributable to trauma sequelae or other conditions such as advanced dementia), insufficient proficiency in the German language, or a refusal to participate. For this study, additional inclusion criteria were age over 18 years (46 patients excluded) and having sustained major injuries (ISS ≥ 9 and requiring intensive care unit (ICU) treatment) (120 patients excluded), resulting in an eligible sample of 515 patients.

### Measures

The primary outcome of the study is a patient-perceived impaired physical function after trauma.

Since impairments are rather subjectively than objectively measurable through clinical methods, we used a patient reported outcome instrument. This is the Trauma Outcome Profile (TOP), which is a validated instrument designed to assess health-related quality of life (HRQoL) in individuals who have experienced major trauma. As a trauma-specific component of the broader Polytrauma Outcome Chart [2, 4], the TOP evaluates 10 distinct domains: depression, anxiety, PTSD, social impact, pain, physical function, daily activities, mental function, body image, and overall satisfaction. For this study, the physical function domain was used which is quantified using a numerical rating scale ranging from 0 (optimal function) to 10 (complete dysfunction). This scale is applied to 14 different body regions. If a body region is rated higher than 0, participants are further queried about the extent to which they are affected by functional limitations using a 5-point Likert scale, from 0 (not at all) to 5 (extremely). The scores for each TOP domain are standardized to a scale of 0 (worst) to 100 (best), with scores of 80 or above reflecting levels comparable to the general population without major trauma [2]. To identify an impaired physical function a value below 80 on the TOP physical function domain was indicative. The same procedure was followed for the TOP pain domain.

### Abbreviated Injury Scale

The Abbreviated Injury Scale (AIS) provides a systematic framework for categorizing and grading the severity of injuries. Developed in the 1960s and refined over time, the AIS assigns severity scores ranging from 1 (minor) to 6 (maximal, actual untreatable) for injuries based on their anatomical location and tissue involvement [1]. This classification system is widely employed in both clinical practice and research to characterize injury patterns and evaluate outcomes across diverse settings. Furthermore, the AIS serves as the foundation for composite scoring systems such as the Injury Severity Score (ISS), facilitating comparisons of trauma severity among different patient populations.

### Injury Severity Score

The Injury Severity Score, introduced in the 1970s, is a widely accepted metric for quantifying overall injury severity in trauma patients [3]. Derived from the AIS, the ISS aggregates the squares of the three highest AIS scores from different body regions. Each body region is rated on a scale from 1 (minor injury) to 6 (life-threatening injury). The resulting ISS ranges from 1 to 75, with higher scores indicating more severe injuries. An ISS of 16 or greater is typically classified as severe trauma, often necessitating intensive care [5]. The ISS is extensively utilized in trauma systems for patient triage and to guide clinical decision-making.

### Statistical analysis

Statistical analyses were carried out using R software (version 4.4.1) [20]. Sociodemographic descriptive characteristics are given as mean with standard deviation (SD) for continuous variables and as frequencies with percentages for categorical variables. To determine relevant predictors regarding an impaired physical function, a logistic regression model was applied. The dependent variable was an impaired physical functional post-trauma, defined as a score below 80 on the 0-100 physical functioning scale of the TOP. Predictors included in the model were age, sex, an impaired physical functionality prior to the trauma, a high overall injury severity scale (defined as ISS ≥ 16), and injuries to specific body areas. The latter included the spinal cord, upper extremities, and lower extremities including the pelvic. This selection was made to avoid collinearity (as all patients have at least 1 affected body area and pre-trauma pain correlates high with physical function) and only those areas that have a high potential for affecting functionality were included. To assess which body area was affected in the accident, an AIS severity level > 1 for the specific area was used. This way, not the severity of the injury was assessed, however only whether a body area was affected at all.

Patients were excluded from the analysis if relevant data about pre-injury and follow-up were incomplete or presented with implausible data (full case analysis). For example, significant decreases in reported physical function regions unrelated to the documented injury pattern were deemed implausible and excluded.

## Results

Among the 515 eligible patients, there were 25 cases with missing data for physical functioning before and/or after the accident, 3 cases without indication of sex, and 7 cases presented implausible data. This resulted in a plausible sample of 480 participants that was used for the final data analysis. On average, the follow-up questionnaires were returned 23 months (SD 3.4 months) after the accident took place. This sample had a mean age of 48.2 years (SD 17.9), with 70.6% of participants being male (Table 1). The most common injury cause was traffic accidents, accounting for roughly 62% of the cases, followed by high falls comprising 19%, and low falls, recorded at 12%. The average ISS was 21.0 (SD 11.1), with 65.6% reporting an ISS ≥ 16. The most affected area (AIS > 1) was the Thorax with 51.3%, followed by the head (45.8%), lower extremities including pelvic (45.2%) and upper extremities (41.0%). About 8% reported an impaired functionality already before trauma and almost 10% reported a relevant pain level.

**Table 1:** Patient characteristics: overall and proportion of patients with post-trauma physical function below cutoff (< 80 out of 100 points). N = 480

	*n*	%
Age		
18–29 years	102	21.2%
30–39 years	56	11.7%
40–49 years	90	18.8%
50–59 years	92	19.2%
60–69 years	75	15.6%
70 years and older	65	13.5%
Gender		
Male	339	70.6%
Female	141	29.4%
Impaired physical function pre-trauma	38	7.9%
Relevant pain pre-trauma	45	9.5%
ISS ≥ 16	315	65.6%
Impacted area (AIS > 1)		
Head	220	45.8%
Thorax	246	51.3%
Abdomen	108	22.5%
Spinal cord	175	36.5%
Upper extremity	197	41.0%
Lower extremity including pelvic	217	45.2%

## Patterns in physical functionality

**Fig. 1:**
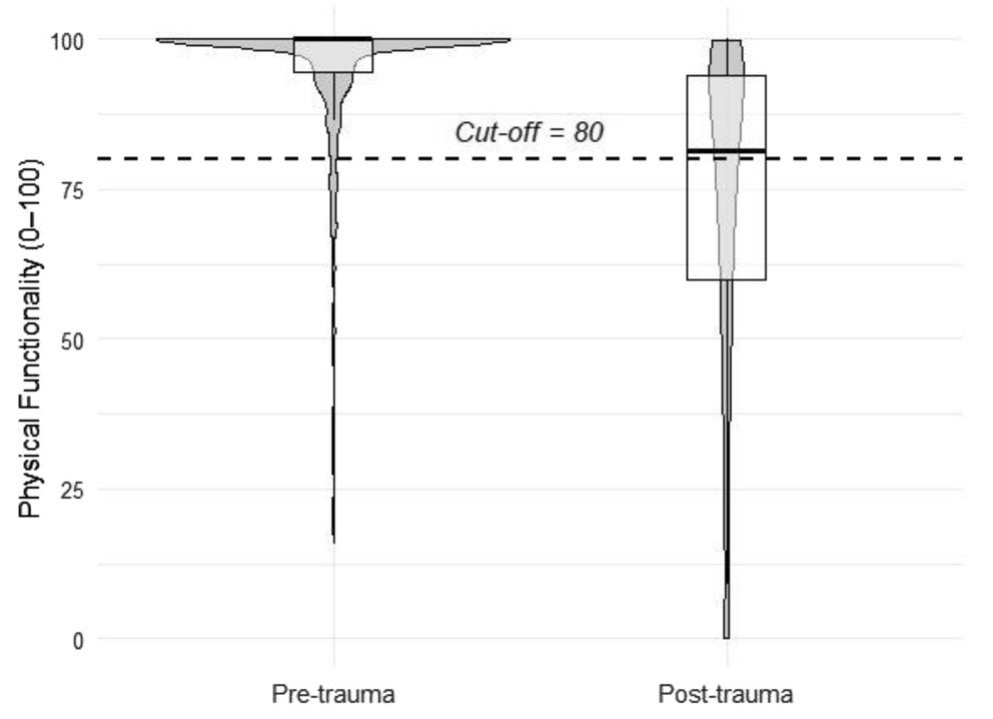
Physical functionality pre- and post-trauma

**Fig. 2:**
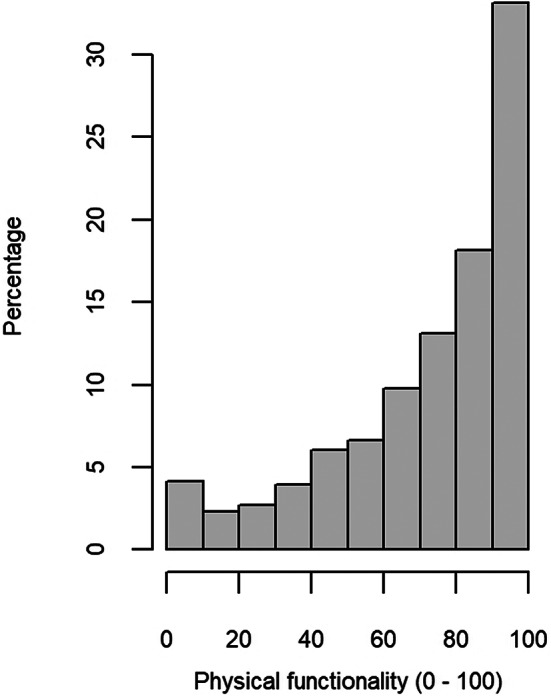
Physical functionality post-trauma

The mean physical function score, where higher values represent better functionality, was 94.8 (SD 11.5) prior to the trauma, with 7.9% (*n* = 38) of patients scoring below the cut-off of 80 points. Post-trauma, the mean score dropped to 72.9 (SD 26.7), with 47.3% (*n* = 227) of patients scoring below the cut-off (Fig. 1). The histogram in Fig. 2 shows the distribution of physical functionality post-trauma in more detail. About a third of the patients experienced almost no limitation (score > 90) in physical functioning. With every 10 points reduction in physical functionality, the number of patients dropped quickly. Almost 20% has a functionality score still over 80, leaving 49% of the patients with a physical functionality score below 80. About 23% is in the range 60–80 and 26% indicated a score even below 60. A small peak can be found in the lower end; about 5% of the patients report the maximum impairment in physical functioning.

**Fig. 3:**
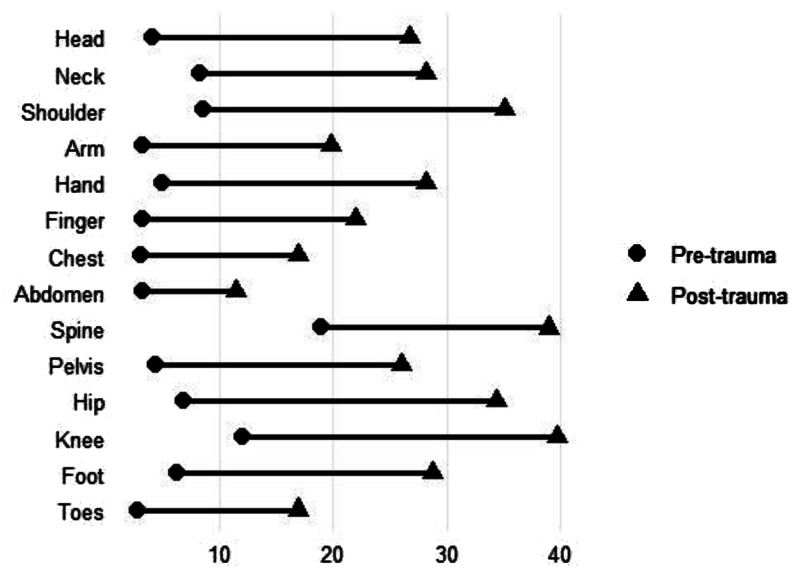
Proportion of patients with moderate to severe limitations (Score > 1) in 14 body regions, pre- and post-trauma

This decline in physical functionality was not only visible on the overall scale; however, each body region showed an increase in patients reporting a reduced physical functioning post-trauma (Fig. 3). The regions that were mentioned most often for having a reduced function (TOP physical function > 1) post-trauma were shoulder, spine, hip, and knee.

An exploratory analysis of treatment-related factors demonstrated that patients with a reduced functionality had a significantly longer hospital stay (median 24 vs. 17 days, *p* < 0.001) and longer ICU stay (median 6 vs. 4 days, *p* = 0.005) compared with patients with a normal functionality. A post hoc exploratory analyses demonstrated a higher proportion of unfavorable functional outcome among patients with psychiatric comorbidity compared with those without psychiatric comorbidity (60.8% vs. 34.9%, χ² test, *p* < 0.001).

### Factors influencing physical functionality

Figures 1 and 3 indicate a clear drop in physical functionality. The logistic regression analysis provides insights in which factors are related to an impaired physical function post-trauma (< 80 points on the TOP physical function scale). The first column in Table 2 shows the unadjusted proportion of patients with physical function below 80.

**Fig. 4:**
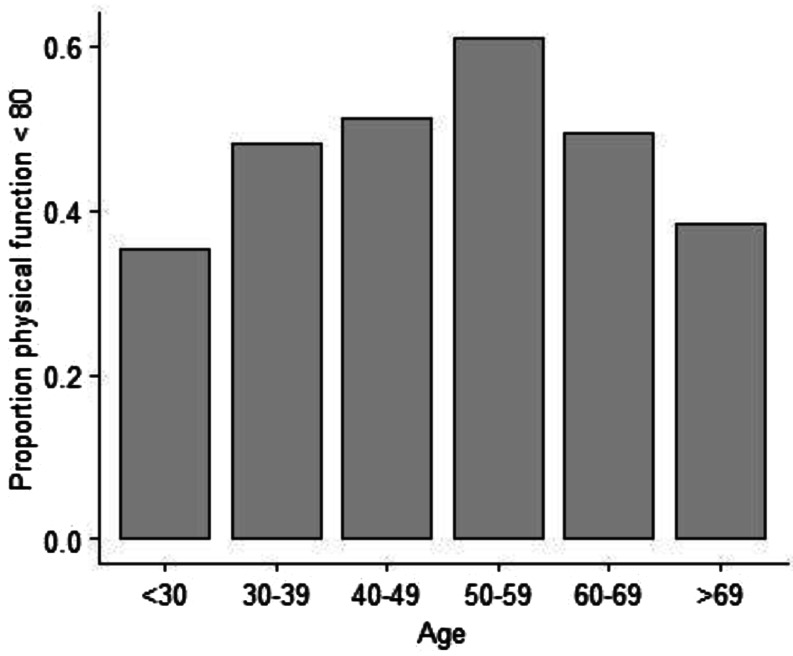
Physical functionality by age

Young adults (aged 18–29) reported with 35.5% the least reduced functionality, whereas the age group between 50 and 59 years showed a peak with 60.9% of the cases reporting an impairment. This non-linear age effect is also illustrated in Fig. 4, clearly showing a peak in the age group 50 and 59 years. Not only young adults show less reduced functionality, interestingly also the oldest age groups indicate less impairments (age 60–69–49.3% and age > 69 38.5%). From the patients with an impaired physical function pre-trauma 86.8% reported an impairment also post-trauma. In case of a severe injury, 51.4% reported an impaired physical function. Patients reported an impaired function, especially when the spinal cord (53.7%), the lower extremities (53.0%), and the upper extremities (52.3%) were involved.

The multivariate logistic regression analysis shows that the most important predictor of post-trauma functional impairment is the pre-trauma physical function. Patients who already had a physical impairment before the trauma happened were eight times more likely (OR 8.21, *p* < 0.001) to also experience a limited functionality after the trauma. The age groups between 30 and 70 were about two times more likely to experience a reduced physical functionality compared to their younger counterparts (age 18–29) with the largest difference for patients aged 50–59 (OR 2.75, *p* = 0.001). Patients aged ≥ 70 were not significant different from the patients aged between 18 and 29 years. Adjusted for baseline characteristics age, sex, pre-trauma physical functionality, the severity of the injury in itself was not significantly related to an impaired physical function (OR 1.49, *p* = 0.060). Even after adjusting for both baseline characteristics and injury severity, the location of the injury was still relevant for recovery. When the lower extremities were affected (AIS > 1), patients were 1.6 times (OR 1.60, *p* = 0.018) more likely to perceive a reduced physical functionality. The spinal cord shows a similar but non-significant effect with an odds ratio of 1.47 (*p* = 0.066). An involvement of the upper extremities was not significantly related to a post-trauma impairment (OR 1.20, *p* = 0.357).

**Table 2:** Logistic regression analysis with physical functioning post-trauma below 80 points as the dependent variable; ref = reference category; McFadden’s R^2^ = 0.089

	Proportion of patients with physical function < 80	Odds Ratio (OR)	95% CI for OR	*P*-value
Age (ref = 18–29 years)	35.3%			
30–39 years	48.2%	1.78	0.90–3.54	0.098
40–49 years	51.1%	1.91	1.04–3.55	0.039
50–59 years	60.9%	2.75	1.50–5.12	0.001
60–69 years	49.3%	1.93	1.02–3.68	0.043
70 years and older	38.5%	1.11	0.56–2.20	0.766
Sex (ref: Male)	45.7%			
Female	51.1%	1.16	0.76–1.77	0.494
Impaired physical function pre-trauma	86.8%	8.21	3.33–24.85	< 0.001
ISS ≥ 16	51.4%	1.49	0.99–2.27	0.060
Impacted area (AIS > 1)				
Spinal cord	53.7%	1.47	0.98–2.20	0.066
Upper extremity	52.3%	1.21	0.81–1.79	0.350
Lower extremity including pelvis	53.0%	1.60	1.09–2.37	0.018

## Discussion

In the present study, we investigated predictors of physical functionality two years after major trauma in a large single-center cohort. The results demonstrate that pre-trauma physical functionality was by far the strongest predictor of long-term outcome. Patients who already experienced physical limitations before the trauma were more than eight times as likely to report functional impairment two years later. This emphasizes the crucial importance of baseline health status and physical reserve in determining recovery potential after major trauma. Interestingly, while overall injury severity was not a significant predictor, injuries involving the lower extremities, including the pelvis, remained robustly associated with poorer physical outcomes. Adults aged 30–69 years showed less favorable recovery than younger adults, whereas no sex differences were observed.

These findings align with previous research highlighting the role of pre-injury functionality and comorbidity in shaping recovery trajectories [7, 13, 19]. Functional recovery after major trauma depends not only on the initial anatomical injury but also on pre-existing musculoskeletal health, fitness, and overall resilience. Patients entering a trauma with lower physical reserves—due to chronic conditions, pain, or limited mobility—may have reduced adaptive capacity and slower rehabilitation progress. The results underscore the necessity of considering pre-trauma functionality as a prognostic factor when designing individualized rehabilitation pathways. In this context, our working group [21] demonstrated that pre-trauma pain itself is a strong independent predictor of persistent enhanced pain patterns after trauma. This finding complements the present results, suggesting that both pre-injury pain and pre-injury functionality represent key risk markers for long-term physical impairment.

Within a study cohort that experienced major trauma (ISS ≥ 9) the non-significant effect of overall injury severity suggests that anatomical damage does not adequately capture the complexity of long-term functional outcomes. This aligns with growing evidence that subjective recovery and functional reintegration are influenced by a complex interplay of biological, psychological, and social factors [11, 17]. Factors such as chronic pain, fatigue, mood disorders, and coping style may moderate the relationship between injury severity and perceived functionality. For example, psychological distress—particularly symptoms of post-traumatic stress and depression—has been shown to exacerbate pain perception and impede motivation and adherence to rehabilitation programs [7]. These mechanisms may explain why two patients with comparable injury patterns can experience vastly different recovery trajectories and suggest that interventions should address these psychosocial domains [7, 17].

The strong impact of lower limb and spinal injuries on long-term functionality is consistent with previous studies demonstrating the enduring effects of these injury sites on mobility, independence, and return-to-work outcomes [15, 18]. Lower extremity trauma often leads to persistent gait abnormalities, muscle weakness, and pain syndromes, while spinal injuries may cause chronic instability and neurological deficits [22–24]. These functional constraints often limit participation in daily activities and contribute to secondary problems such as deconditioning and psychological distress. Tailored physiotherapeutic programs focusing on mobility restoration, postural control, and pain management may therefore be particularly beneficial for these groups.

The age-related differences in recovery are also noteworthy. Adults in midlife (30–64 years) reported significantly more limitations than younger adults, whereas outcomes among the oldest participants (≥ 65 years) did not differ significantly from the youngest group. This somewhat unexpected pattern could reflect competing influences: while younger patients may have greater biological resilience and motivation for recovery, middle-aged adults may face higher psychosocial burdens, such as occupational and familial responsibilities, which can impede full rehabilitation engagement. Moreover, this age group may have more pre-existing musculoskeletal wear or comorbidities, despite being younger than the traditionally “elderly” population. These findings suggest the need for age-sensitive rehabilitation approaches that account for differences in physical and psychosocial contexts.

The inability to include pain as a predictor in the regression model due to collinearity reasons emphasizes the interconnectedness between pain and functional impairments. Previous research—including the recent work [21]—demonstrates that pre-existing and persistent pain are among the most influential determinants of functional limitation and quality of life after trauma. Pain and functional impairment reinforce each other bidirectionally: reduced activity can worsen pain perception and muscle atrophy, while chronic pain reduces mobility, self-efficacy, and emotional well-being [8, 14]. Multimodal pain management strategies, including early psychological support, could be considered to improve long-term recovery.

This study’s strengths include its large sample size, standardized assessment using a validated trauma-specific patient-reported outcome instrument (Trauma Outcome Profile), and the relatively long follow-up period of nearly two years. Using patient-reported functionality provides valuable insights into how individuals perceive their recovery—an aspect often missed by purely clinical metrics. However, several limitations should be acknowledged. The single-center design may limit generalizability to other settings, and selection bias cannot be excluded. An important methodological consideration is the retrospective assessment of pre-trauma physical functionality. In trauma outcome research, prospective baseline assessment prior to injury is generally not feasible, making retrospective self-report a commonly applied and accepted approach. Watson et al. demonstrated that retrospective baseline assessment is particularly suitable for physical and role functioning domains, although some degree of recall bias remains unavoidable [25]. In addition, more recent trauma outcome research confirmed that pre-injury health status represents one of the most relevant prognostic factors for long-term recovery after injury [26]. Nevertheless, patients may partially base their retrospective assessment on their current health status, potentially leading to an overestimation of the difference between pre- and post-trauma functionality. Therefore, the present findings regarding pre-trauma functionality should be interpreted with appropriate caution.for this study Moreover, the reliance on self-reported measures introduces subjectivity, which—although relevant for understanding patient experience—may differ from objective clinical assessments. Furthermore, the use of AIS > 1 only allowed identification of affected body regions, but did not differentiate between specific injury patterns or severity within these regions. Therefore, clinically important differences between injuries affecting the same body area may not have been fully captured. Patients with severe cognitive impairment who were unable to complete the questionnaire were not represented in this cohort. The study population may underrepresent patients with the highest degree of long-term impairment, which should be considered when interpreting the observed association between injury severity and long-term functionality. In addition, information regarding whether injuries were work-related was not available for the present analysis. Differences in compensation systems, occupational demands, and return-to-work support may influence long-term functional recovery and should be considered in future studies. Future studies should combine subjective and objective outcome measures, such as performance-based physical tests, to capture recovery more comprehensively.

## Conclusions

The findings underline the importance of assessing pre-trauma baseline functionality and health status early in the rehabilitation process. Patients with pre-existing impairments, as well as those with spinal or lower limb injuries, should receive intensified, multidisciplinary rehabilitation including physical, psychological, and social components. Early identification of these risk groups may allow healthcare professionals to set realistic recovery goals and allocate rehabilitation resources more effectively. Furthermore, integrating routine psychological assessment and multimodal pain management into trauma aftercare could substantially improve long-term outcomes.

Future research should investigate how physical, social, and psychological factors interact over time to influence recovery. Longitudinal, multi-center studies combining registry data with patient-reported outcomes could help disentangle these complex relationships. This also allows to test the effect of baseline functionality in which the baseline functionality is collected closer in time to the trauma. The development of predictive models that include demographic, clinical, and psychosocial variables may support personalized rehabilitation planning and improve overall post-trauma quality of life.

## Data Availability

The datasets used and analyzed during the current study are available from the corresponding authors on reasonable request.

## Abbreviations

ISS: Injury Severity Score
TOP: Trauma Outcome Profile
AIS: Abbreviated Injury Scale
HRQoL: Health-related quality of life
PTSD: Post-traumatic stress disorder
ICU: Intensive Care Unit
SD: Standard Deviation
OR: Odds Ratio
CI: Confidence Interval
GOS: Glasgow Outcome Scale

## References

[1] Haasper C, Junge M, Ernstberger A, Brehme H, Hannawald L, Langer C, Nehmzow J, Otte D, Sander U, Krettek C. The Abbreviated Injury Scale (AIS. Options and Problems in Application. Unfallchirurg. 2010;Bd(113):366–72. 10.1007/s00113-010-1778-8 20376615

[2] Lefering R, Tecic T, Schmidt Y, Pirente N, Bouillon B, Neugebauer E, Group P. Quality of Life after Multiple Trauma: Validation and Population Norm of the Polytrauma Outcome (POLO) Chart. Eur J Trauma Emerg Surg. 2012;38:403–15. 26816121 10.1007/s00068-011-0149-7

[3] Linn S. The Injury Severity Score—Importance and Uses. Ann Epidemiol Bd. 1995;5:440–6. 10.1016/1047-2797(95)00059-3 8680606

[4] Pirente N, Ottlik Y, Lefering R, Boullion B, Neugebauer E, Group W. (2006). Polytrauma. The DGU*, Bd. 2006;32:44–62.

[5] Stevenson M, Segui-Gomez M, Lescohier I, Scala C, McDonald-Smith G. An Overview of the Injury Severity Score and the New Injury Severity Score. Inj Prev Bd. 2001;7:10–3. 10.1136/ip.7.1.10 PMC1730702 11289527

[6] Frydrych LM, Keeney-Bonthrone TP, Gwinn E, Wakam GK, Anderson MS, Delano MJ. Short-term versus long-term trauma mortality: A systematic review. J Trauma Acute Care Surg. 2019;87(4):990–7. 10.1097/TA.0000000000002430. 31589196 10.1097/TA.0000000000002430

[7] Gabbe BJ, Simpson PM, Cameron PA, Ponsford J, Lyons RA, Collie A, Fitzgerald M, Judson R, Teague WJ, Braaf S, Nunn A, et al. Long-term health status and trajectories of seriously injured patients: a population-based longitudinal study. PLoS Med. 2017;14(7). 10.1371/journal.pmed.1002322 10.1371/journal.pmed.1002322 PMC5497942 28678814

[8] Kaske S, Lefering R, Trentzsch H, Driessen A, Bouillon B, Maegele M, Probst C. Quality of life two years after severe trauma: A single centre evaluation. Injury. 2014;45:100–5. 10.1016/j.injury.2014.08.028 25284226

[9] Kruithof N, Polinder S, de Munter L, van de Ree CLP, Lansink KWW, de Jongh MAC, BIOS-group. Health status and psychological outcomes after trauma: A prospective multicenter cohort study. PLoS ONE. 2020;15(4):e0231649. 10.1371/journal.pone.0231649. 32315373 10.1371/journal.pone.0231649 PMC7173764

[10] Holbrook TL, Anderson JP, Sieber WJ, Browner D, Hoyt DB. Outcome after major trauma: 12-month and 18-month follow-up results from the Trauma Recovery Project. J Trauma. 1999;46(5):765–71. 10.1097/00005373-199905000-00003. 10338392 10.1097/00005373-199905000-00003

[11] Meakes S, Enninghorst N, Weaver N, Hardy BM, Balogh ZJ. Long-term functional outcomes in polytrauma: A fundamentally new approach is needed in prediction. Eur J Trauma Emerg Surg Bd. 2024;50(4):1439–52. 10.1007/s00068-023-02430-6 PMC11458641 38358513

[12] Vles WJ, Steyerberg EW, Essink-Bot M-L, van Beeck EF, Meeuwis JD, Leenen LPH. Prevalence and determinants of disabilities and return to work after major trauma. J Trauma. 2005;58(1):126–35. 10.1097/01.ta.0000112342.40296.1f. 15674163 10.1097/01.ta.0000112342.40296.1f

[13] Berger-Estilita J, Granja C, Gonçalves H, Dias CC, Aragão I, Costa-Pereira A, Orwelius L. A new global health outcome score after trauma (GHOST) for disability, cognitive impairment, and health-related quality of life: Data from a prospective cross-sectional observational study. Brain Injury Bd. 2019;33(7):922–31. 10.1080/02699052.2019.1581257 30810390

[14] Duckworth MP, Iezzi T. Motor Vehicle Collisions and Their Consequences—Part II: Predictors of Impairment and Disability. Psychol Injury Law Bd. 2018;11(3):288–306.

[15] Neubert A, Hempe S, Jaekel C, Gaeth C, Spering C, Fetz K, Windolf J, Kollig E, Bieler D. Lived experiences of working-age polytrauma patients in Germany—A qualitative Analysis. Injury. 2025;Bd(56):1. 10.1016/j.injury.2024.111938 39477709

[16] Simmel S, Bühren V. Polytrauma überlebt – und was kommt dann? Der Unfallchirurg Bd. 2009;112(11):965–74. 10.1007/s00113-009-1686-y 19816668

[17] Wurm S, Röse M, Rüden C, Woltmann A, Bühren V. Das schwere Polytrauma mit einem ISS ≥ 50. Z für Orthopädie und Unfallchirurgie Bd. 2012;150(03):296–301. 10.1055/s-0031-1280415 22328201

[18] Janssen C, Ommen O, Neugebauer E, Lefering R, Pfaff H. Predicting Health-related Quality of Life of Severely Injured Patients: Sociodemographic, Economic, Trauma, and Hospital Stay-related Determinants. Eur J Trauma Emerg Surg Bd. 2008;34(3):277–86. 10.1007/s00068-008-7054-8 26815750

[19] Holtslag HR, Beeck EF, Lindeman E, Leenen LPH. Determinants of Long-Term Functional Consequences After Major Trauma. J Trauma: Injury Infect Crit Care Bd. 2007;62(4):919–27. 10.1097/01.ta.0000224124.47646.62 17426549

[20] R Core Team. R: A Language and Environment for Statistical Computing. Vienna, Austria: R Foundation for Statistical Computing; 2024. https://www.R-project.org/.

[21] Fetz K, Lefering R, Kaske S. Pre-Trauma Pain Is the Strongest Predictor of Persistent Enhanced Pain Patterns after Severe Trauma: Results of a Single-Centre Retrospective Study. Medicina. 2023;59(7):1327. 10.3390/medicina59071327. 37512138 10.3390/medicina59071327 PMC10383629

[22] Amanat M, Vaccaro AR, Salehi M, Rahimi-Movaghar V. Neurological conditions associated with spinal cord injury. Inf Med Unlocked. 2019;16:100245. 10.1016/j.imu.2019.100245.

[23] Pfeifer R, Zelle BA, Kobbe P, Knobe M, Garrison RL, Ohm S, Sittaro N-A, Probst C, Pape H-C. Impact of isolated acetabular and lower extremity fractures on long-term outcome. J Trauma Acute Care Surg. 2012;72(2):467–72. 10.1097/TA.0b013e318219fbfa. 22439211 10.1097/ta.0b013e318219fbfa

[24] Rauer T, Friedl E, Gamble JG, Zelle BA, Pape H-C, Pfeifer R. Long-term analysis of chronic pain associated with lower extremity injuries. Arch Orthop Trauma Surg. 2022;143(7):4149–54. 10.1007/s00402-022-04717-6. 36454306 10.1007/s00402-022-04717-6 PMC10293374

[25] Watson WL, Ozanne-Smith J, Richardson J. Retrospective baseline measurement of self-reported health status and health-related quality of life versus population norms in the evaluation of post-injury losses. Inj Prev. 2007;13(1):42–5. 10.1136/ip.2006.012138. 10.1136/ip.2005.010157 PMC2610562 17296689

[26] de Munter L, Polinder S, Havermans RJM, et al. Prognostic factors for recovery of health status after injury: a prospective multicentre cohort study. BMJ Open. 2021;11:e038707. 10.1136/bmjopen-2020-038707. 10.1136/bmjopen-2020-038707 PMC7789437 33408198

